# Endophthalmitis With Subretinal Abscesses Presenting as Charles Bonnet Syndrome in a Patient With Methicillin-Resistant Staphylococcus aureus (MRSA) Bacteremia: A Case Report and Discussion of the Literature

**DOI:** 10.7759/cureus.85737

**Published:** 2025-06-10

**Authors:** Leanna M Zelle, Jeffrey L Bodeen, Jonah Shah, Vaishnavi Balendiran, Ahmed Elkeeb

**Affiliations:** 1 Ophthalmology, University of Missouri, Columbia, USA

**Keywords:** bacterial endogenous endophthalmitis, charles bonnet syndrome, intraocular antibiotics, mrsa bacteremia, post-operative delirium, septic arthritis of knee, serous retinal detachment, subretinal abscess

## Abstract

We present the case of a 73-year-old male with recurrent necrotizing fasciitis and methicillin-resistant Staphylococcus aureus (MRSA) bacteremia who developed visual hallucinations and altered mental status on post operative day one after undergoing right knee prosthetic joint infection washout. His visual acuity, which was self reported as previously “very good” decreased to hand motion in the right eye (OD) and 20/400-1 in the left eye (OS), and he described new visual hallucinations. Differentials included post operative delirium, stroke, and ocular infiltration of his bacteremia. He was evaluated by infectious disease, orthopedic surgery, neurology, and ophthalmology specialties, and was found to have bilateral endophthalmitis, Charles Bonnet syndrome, and bilateral subretinal abscesses. The patient was treated with intravenous (IV) antibiotics and surgical removal of his infected knee hardware; however, he and his family members ultimately made the decision to withdraw goal-directed care. We present this case to emphasize the importance of ophthalmologic evaluation and early intervention in patients with MRSA bacteremia and alterations in vision or mentation.

## Introduction

Endophthalmitis is a rare complication of bacteremia, and it is typically exogenous secondary to cataract surgery, intravitreal injection, blebitis, or penetrating eye trauma [1]. However, endophthalmitis can also occur without insult to the anterior ocular structures through means of bacteremia or fungemia, termed endogenous endophthalmitis. Bacterial endogenous endophthalmitis often presents in patients with bacterial meningitis, pyogenic arthritis, internal organ abscesses, osteomyelitis, prostatitis, and vascular indwelling catheters [1]. Patients with endogenous endophthalmitis typically present with decreased vision, visual field changes, and eye pain. Patients can develop complications from endophthalmitis, including visual loss. One rare complication of endophthalmitis-related visual loss is Charles Bonnet syndrome, which describes visual hallucinations following great decreases in visual acuity [2]. Another rare finding, subretinal abscesses, can occur and are associated with irreversible visual loss. Optimal treatment of retinal abscesses has not yet been determined [3,4]. We present the case of a 73-year-old male with recurrent necrotizing fasciitis and methicillin-resistant Staphylococcus aureus (MRSA) bacteremia who developed visual hallucinations and altered mental status on postoperative day one after undergoing right knee prosthetic joint infection washout and was found to concurrently have bilateral endophthalmitis, Charles Bonnet syndrome, and bilateral retinal abscesses.

## Case presentation

A 73-year-old male patient presented to an outside emergency department (ED) with a three-week history of increasing right leg and back pain. He had a complex past medical history including: insulin dependent type II diabetes mellitus complicated by neuropathy and foot ulcers; MRSA bacteremia; necrotizing fasciitis of the left lower extremity (LLE) requiring left above-the-knee amputation (AKA); subsequent necrotizing fasciitis of the right upper extremity; Charcot foot; non-alcoholic steatohepatitis; recent LLE deep venous thrombosis on apixaban; and obstructive sleep apnea. Following his hospitalizations for necrotizing infections and MRSA bacteremia secondary to diabetic foot ulcers, he was treated outpatient with vancomycin for eight weeks and doxycycline for an additional four weeks. He completed this antimicrobial therapy six weeks prior to his presentation. 

His surgical history was remarkable for bilateral total knee arthroplasty 11 years prior to presentation and LLE AKA four months prior to presentation. He denied alcohol, tobacco, intravenous drug use, or other illicit substance use. He had no past ophthalmic history. 

In the ED, laboratory evaluation was significant for leukocytosis of 18 (reference range 3.5-10.5). He was diagnosed with L3 vertebral compression fracture shown on CT spine and acute kidney injury (AKI), given fentanyl for pain control, and discharged home with an orthopedic follow-up. 

**Figure 1:**
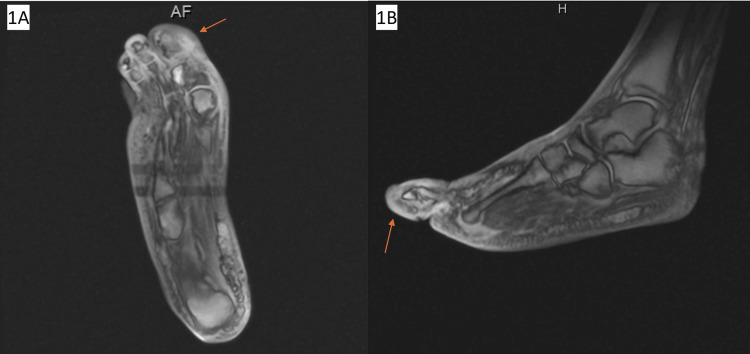
MRI of the right lower extremity in the axial plane (A) and sagittal plane (B) showing diffuse subcutaneous edema consistent with cellulitis (red arrow), but no signs of osteomyelitis.

Three days later, he presented to an outside ED with continued pain and fever. Vitals were significant for temperature of 38.3 degrees C and tachycardia in the 150s. EKG revealed atrial fibrillation (AFib) with rapid ventricular response (RVR). He was transferred and directly admitted to the family medicine service of an academic institution for new-onset AFib with RVR, fever, and AKI. On physical exam, he had pitting edema to the right thigh with no warmth or erythema. MRI of the right lower extremity (RLE) showed diffuse subcutaneous edema of the foot consistent with cellulitis, but no osteomyelitis (Figure 1). Chest X-ray was unremarkable. Laboratory evaluation was significant for elevated inflammatory markers with erythrocyte sedimentation rate (ESR) 52 mm/Hr (reference range 0.0-20.0 mm/Hr) and C-reactive protein (CRP) of 41.05 mg/dL (reference range 0.00-0.50 mg/dL). Four blood cultures were drawn, and all resulted positive for MRSA. The patient was started on IV vancomycin. Echocardiogram was ordered to rule out endocarditis, and the infectious disease (ID) team was consulted. 

On hospital day two, he fevered to 39.2 degrees Celsius and developed a headache and increased right knee pain. Orthopedic surgery performed a washout of the right knee due to concern for prosthetic joint infection. His pain was managed with IV Tylenol as the patient did not want any opioid analgesics. 

On hospital day three, the patient reported concerns that his pain medications were making him hallucinate. The primary team discussed potential etiologies of mental status change with the patient and his family, including recent surgery and sedation, MRSA bacteremia, and delirium. The patient denied visual changes at the time of morning rounds. Infectious disease saw the patient about an hour later, and patient then endorsed visual changes such as feeling like he was seeing through "an aquarium" or looking through "a forest.” The ophthalmologist was consulted, and MRI brain was ordered. Then, approximately an hour after, the patient's nurse called the primary team due to concerns of acute right-sided visual loss and concern for a stroke. On initial evaluation, the patient was alert and oriented to person, place, and time. He reported that things looked like "swirling concrete" and was unable to focus on a point in space. He reported blurry, dark vision in his right eye, and was unable to see in his periphery. He was unable to complete finger-to-nose or visual field testing successfully due to his confusion and inability to follow commands. Due to his inability to complete the initial testing, there was a high concern for a stroke and code stroke was promptly called. 

**Figure 2:**
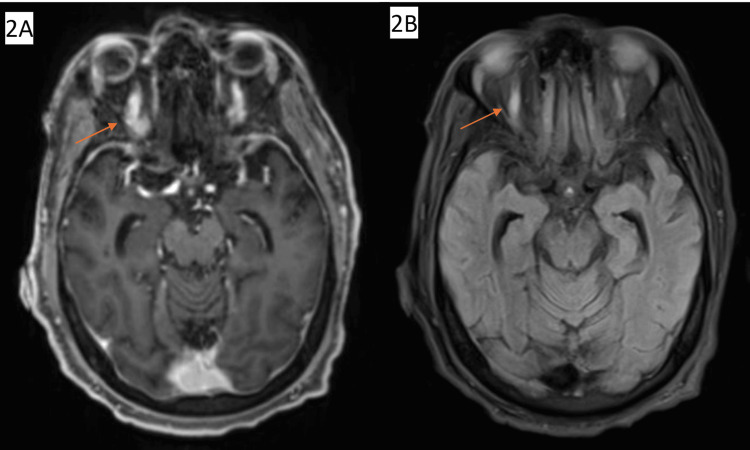
MRI Brain (A, B) in the axial plane showing right superior ophthalmic vein enlargement.

MRI brain showed no evidence of stroke but was significant for a few nonspecific scattered punctate foci of increased T2 fluid attenuated inversion recovery (FLAIR) signal intensity in the subcortical white matter likely related to diabetes or hypertension, mild indeterminate pachymeningeal thickening, asymmetric fullness of the left cavernous sinus, and likely incidental microadenoma. There were no changes in the orbital contents. The right superior ophthalmic vein was slightly thicker than the left (Figure 2). Vascular neurology determined that ischemia was an unlikely etiology for this patient’s sudden-onset visual changes. 

On ophthalmology consultation, the patient reported that he was seeing "birds flying through the foliage.” He subsequently reported seeing an octopus moving around in his vision. He denied double vision, flashes/floaters/curtains, or eye pain. He reported no past ophthalmologic problems or surgeries. His vision was self-reported to have been very good prior to these recent changes. His family history was negative for glaucoma, macular degeneration, or retinal detachment. His visual acuity was hand motion in the right eye (OD) and 20/400-1 in the left eye (OS). His intraocular pressures (IOPs) were normotensive at 15 mmHg OD and 11 mmHg OS (reference range 10-21 mmHg). His pupils were reactive with no relative afferent pupillary defect (RAPD). Extraocular movements (EOM) were full in both eyes (OU). Anterior segment exam showed no abnormalities. There was a poor view on the dilated fundus exam (DFE) due to vitreous debris. A white lesion nasal to the nerve was visible OS. 

**Figure 3:**
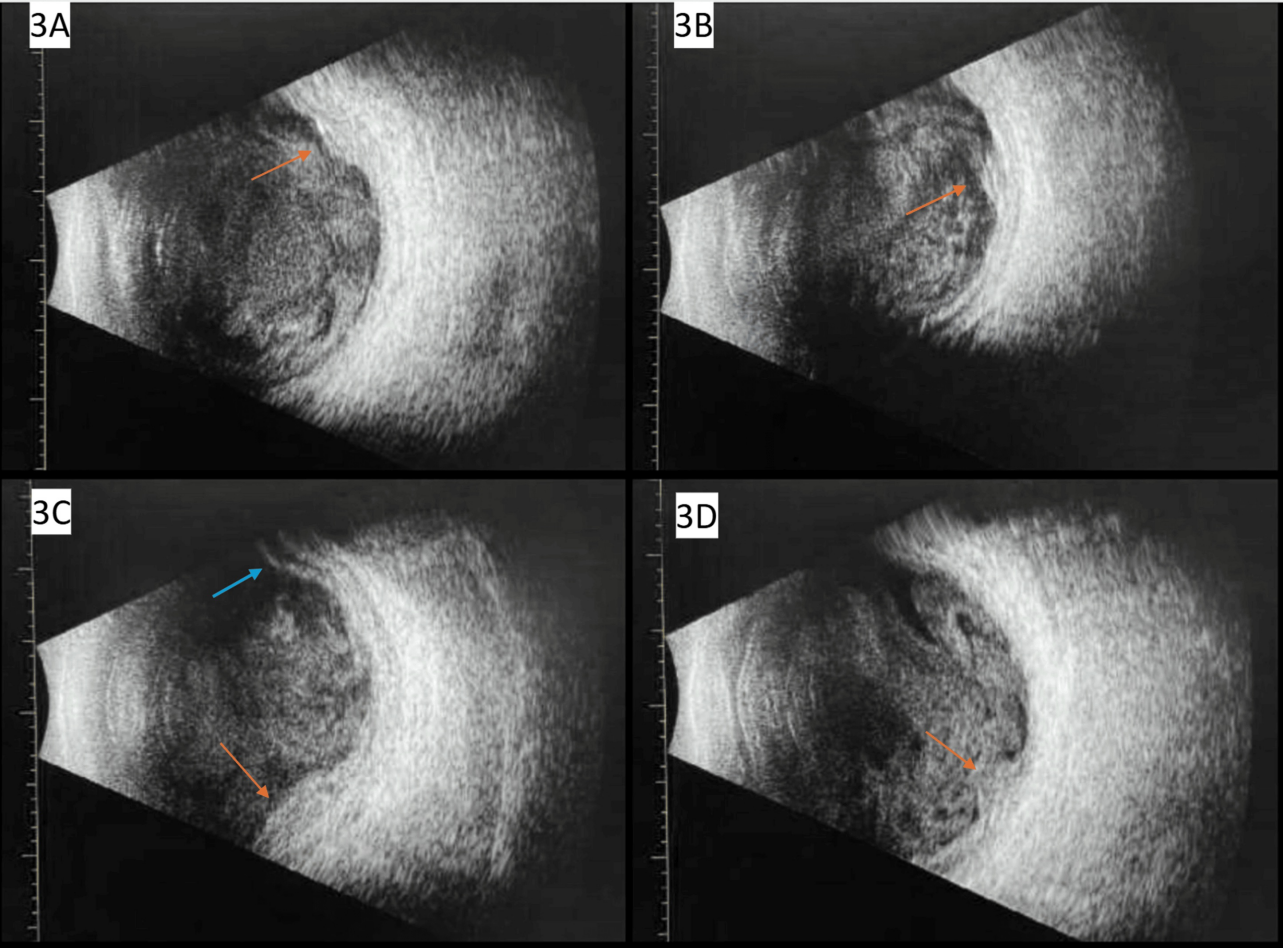
A, B, C: B-scan OD showing vitreous debris, multiple subretinal abscesses (red arrows), focal serous retinal detachment (blue arrow). D: B-scan showing OS vitreous debris and subretinal abscess (red arrow). OD: right eye, OS: left eye

B-scan ultrasonography was performed (Figure 3), which showed vitreous debris OU, subretinal abscesses OU, and a serous retinal detachment OD. The ophthalmologist diagnosed him with endogenous endophthalmitis OU, subretinal abscesses OD worse than OS, serous retinal detachment OD, and Charles Bonnet syndrome in the setting of known MRSA bacteremia. IV vancomycin was continued in addition to prednisolone acetate 1% eye drops every six hours OU to reduce intraocular inflammation. Given the endogenous source and extensive bilateral disease, ophthalmologic surgical intervention was not recommended at this time. The ophthalmologist planned for close follow-up to monitor for complications like retinal tears or tractional membrane formation.. 

The patient subsequently underwent escalation of his antimicrobial treatment with daptomycin and ceftaroline, blood transfusion due to worsening anemia, and additional debridement and removal of his right knee hardware. The accumulation of several debilitating comorbidities led our patient to withdraw disease-oriented care after discussion between the patient, family, and care team. The patient was discharged to home hospice on lorazepam 0.5 mg every hour as needed, quetiapine 25 mg nightly, gabapentin 300 mg twice daily, hyoscyamine 0.125 mg every hour as needed, morphine 5 mg every hour as needed, and oxycodone every four hours as needed. The patient did not follow up with our health system following hospital discharge. 

## Discussion

Endophthalmitis is defined as inflammation of the internal structures of the eye due to infection. Endophthalmitis is typically exogenous, where an infective agent enters the eye from an external source, frequently occurring secondary to cataract surgery, intravitreal injection, blebitis, or punctuating eye trauma [1]. Endogenous endophthalmitis, however, occurs when the infection spreads hematogenously to the eye from an extraocular source. In this patient's case, he had a history of MRSA bacteremia that was treated outpatient, but recurred. We suspect that his knee hardware became infected during a prior episode of MRSA bacteremia, and this was the reason his outpatient antibiotic regimen was not sufficient to clear the infection. As remaining bacteria were likely rooted in his infected knee hardware, we suspect this led to a recurrent bacteremia and subsequently seeded to the choroid in both eyes. 

The incidence of bacteremia in hospitalized patients is approximately one in 2000, with bacteremia and fungemia being the most common causes of endogenous endophthalmitis [1]. Frequent sources of bacteremia that lead to endogenous endophthalmitis include bacterial meningitis, pyogenic arthritis, internal organ abscesses, osteomyelitis, prostatitis, and vascular indwelling catheters [1]. MRSA and Streptococcus are the most commonly identified species in endogenous endophthalmitis patients [1,5]. Given this patient's history of hospitalization with surgical debridement and amputation prior to this admission, his bacteremia is most likely nosocomial in origin. With his MRSA bacteremia, this is expected as there are higher rates of methicillin-resistant than methicillin-sensitive S. aureus in hospitalized patients [6]. 

A notable complication of the patient’s endogenous endophthalmitis is the development of bilateral subretinal abscesses. The two most prevalent symptoms in subretinal abscesses are changes in visual acuity, occurring in approximately 70% of patients, and eye pain, present in 30-65% of patients [3]. This patient presented with changes in visual acuity but no pain, however he still underwent ocular ultrasound (B-scan) which revealed the presence of subretinal abscesses. Subretinal abscesses are an uncommon complication of endogenous endophthalmitis. This is a sight-threatening infection where a pathogen reaches the choroid via the bloodstream and crosses the blood-retinal barrier, invading the retina and potentially the vitreous cavity [7]. The etiology of subretinal abscess can be classified into two domains. The first of the two domains is a subretinal abscess without identified systemic foci. The two most common pathogens in this classification, per a 2022 review of 105 articles, are Aspergillus and Nocardia [3]. The second classification is a subretinal abscess with identifiable foci, which is the classification of the patient presented in this case. The most common pathogens identified in patients with subretinal abscesses having identifiable foci are Nocardia, Mycobacterium tuberculosis, Klebsiella, and S. aureus [3,4]. The most common risk factors seen in both categories of subretinal abscesses are immunosuppression and diabetes mellitus [3]. We suspect poorly controlled diabetes mellitus as the most prominent risk factor for this patient, as he had additional end-organ effects of diabetes including Charcot foot and diabetic wounds requiring amputation. 

There are no current official guidelines for treating subretinal abscesses, but there are mainstays in treatment: intravitreal antibiotics/antifungals, systemic antibiotics/antifungals, and pars plana vitrectomy. In patients without systemic foci, vitrectomy is the first-line treatment. In patients with foci, systemic antibiotics and intravitreal antibiotics are first-line, and surgical options are pursued when non-surgical options have failed [3]. Our patient underwent surgical debridement of the knee by the orthopedic surgery team, and remained on IV antibiotic therapy, without intravitreal antibiotic administration or vitrectomy. Since our patient made the decision to withdraw goal-directed care, it is unknown if the IV antibiotics would have controlled his endophthalmitis and improved his visual acuity, or if the ophthalmologist would have advanced to the more invasive treatments such as intravitreal injection or vitrectomy. It remains unknown which treatment options would have been successful for this patient or what his visual outcome would have been. Additionally, to avoid the risk of worsening inflammation, bleeding, or new infection associated with penetrating the ocular tissue, our patient did not undergo ocular fluid sampling, which would have been a helpful confirmatory diagnostic test. This, along with the lack of long-term follow-up data on this patient, are limitations of this case. 

The prognosis of subretinal abscesses improves significantly with the prompt initiation of systemic antibiotics in cases without vitreous involvement. However, the treatment is more controversial in cases with vitreous involvement. The effectiveness of certain antibiotics is also a matter of debate, given the varying penetrance of antibiotics across the blood-retinal barrier. There are not currently any randomized controlled trials that provide sufficient evidence for any particular antibiotic choice in endophthalmitis. 

One literature review assessed various studies that include data from rabbit studies, human studies, case reports, and studies evaluating CSF penetration of systemic antibiotics [8]. Due to the lack of similar study designs, the conclusions are not generalizable to all patients. The choice of antibiotics in patients with endophthalmitis becomes individual to the patient’s specific clinical scenario. This literature review concluded that the antibiotics likely to have good blood retina barrier penetration include meropenem, linezolid, and moxifloxacin. The antibiotics that show some potential for retinal penetration, but possible inability to reach therapeutic levels, include vancomycin, cefazolin, ceftriaxone, ceftazidime, imipenem, daptomycin and trimethoprim/sulfamethoxazole. In this patient’s case, MRSA bacteremia requires vancomycin, regardless of its ability to penetrate the blood retinal barrier or reach therapeutic concentrations. Likewise, in any case of endogenous endophthalmitis, it is common to use systemic antibiotics as a systemic infection led to the ocular involvement. The review concluded that antibiotics with poor retinal penetration include ciprofloxacin, levofloxacin, aminoglycosides, aminopenicillins, piperacillin, cefepime and clarithromycin. However, these conclusions do remain controversial given the lack of randomized controlled trials and meta-analyses on this topic. Further research needs to be conducted before strong recommendations on systemic antibiotic therapy can be made for any type of endophthalmitis, including exogenous, endogenous, or cases complicated by retinal abscesses. Thus, topical and intraocular administration of antibiotics remain the most common methods of antibiotic delivery for patients with endophthalmitis [8]. 

Surgical treatment, including pars plana vitrectomy and abscess drainage, is typically considered a second-line therapy when non-surgical options are not effective. However, some ophthalmologists and researchers advocate early surgical intervention for very aggressive pathogens. Some have adopted the technique of intralesional antibiotics, namely the injection of antibiotics into the subretinal space through a small retinotomy. Compared to abscess drainage, there is some evidence that intralesional injections have a decreased risk of retinal detachment [9]. 

Patients with endogenous endophthalmitis typically present with decreased vision, visual field changes, and eye pain [10]. Though this patient did present with decreased vision, his case is atypical in that he did not experience any pain. On the third day of hospitalization, the patient developed visual hallucinations and was diagnosed with Charles Bonnet syndrome following the ophthalmology consult; in concurrence with bilateral endogenous endophthalmitis, subretinal abscesses bilaterally more severe in the right eye, and serous retinal detachment as shown on the B-scan. Differentials for his symptoms remain broad, including cerebrovascular events, optic neuropathy, bacterial or fungal endophthalmitis, posterior uveitis, inflammatory or autoimmune conditions such as sarcoidosis, or neoplasm. However, given his clinical history of bacteremia, his brain MRI, and ocular exam, MRSA endogenous endophthalmitis remains the most likely etiology for his symptoms. His diagnoses can explain the origin of vision loss; combined with the diagnosis of Charles Bonnet syndrome, this offers an explanation of the visual hallucinations he experienced. 

Charles Bonnet syndrome, also known as visual release hallucinations, is a collection of symptoms, primarily visual hallucinations, following vision loss or decrease in visual acuity and occurring without a psychiatric cause [1,2,11]. One study has shown that 11 to 15% of patients with vision loss report some sort of visual hallucinations [11]. However, in current research, it is hypothesized to be more common than currently present in literature as patients do not report this symptom to family or providers for fear of being diagnosed with a psychiatric illness [11]. Patients with more severe vision loss report higher rates of visual hallucinations [11]. The age range of patients presenting with Charles Bonnet syndrome is vast. Cases report visual release hallucinations in children as young as six years old, but it is markedly more common in older adults, likely due to vision-threatening conditions occurring more frequently in adults compared to children [12,13]. 

Common causes leading to the development of Charles Bonnet syndrome include age-related macular degeneration, glaucoma, diabetic retinopathy, and cerebral infarction [2,10-14]. However, any lesion in the visual pathway can lead to the development of visual release hallucinations. The most widely accepted explanation is that hallucinations occur when visual sensory deafferentation leads to disinhibiting visual cortical regions and hyperexcitability with spontaneous neuronal activity [14]. Some of the possible risk factors for Charles Bonnet syndrome are cognitive impairment, cerebral vascular disease, and cortical atrophy of the brain [15]. 

This patient and his family made the decision to withdraw goal-directed care before he could receive further work-up or treatment of his retinal abscesses. Given the patient's already atypical presentation, it is difficult to speculate the clinical course and visual outcome the patient would have had if he had chosen to receive further goal-directed care. Treatment for Charles Bonnet syndrome is individualized to the patient's needs. Initial treatment is reassurance and education about the nature of the syndrome. Notably, if the vision loss is corrected, the symptoms may improve. However, regardless of how well visual acuity can be regained, 75% of patients report symptom duration of visual hallucinations beyond five years [16]. There are also countless conditions that result in irreversible visual loss, and the patient’s visual hallucinations are more likely to remain. Some negative prognostic factors for our patient include poor visual acuity and highly virulent bacteremia, which is the source of endophthalmitis [10,15,17]. Despite these poor prognostic factors, it is impossible to predict if our patient would have regained vision or not. 

Endophthalmitis is a rare complication of bacteremia, and retinal abscesses are a rare complication of endophthalmitis. In these patient populations, it is helpful to have an understanding of their psychiatric and neurologic baseline, especially when evaluating them for acute visual field loss. Patients with low visual acuity report lower rates of visual release hallucinations when they are less isolated, thus highlighting the role for a supportive, psychosocial approach. With the support of family, his primary care team, and his consulting physicians, this patient chose to leave the hospital and continue symptom-directed care at his home. Though there were theoretical treatments yet to be attempted, the most important part of this patient’s care is that he was able to choose the plan that was right for him. Palliative care physicians provide invaluable insight into a patient’s end-of-life care plans, and those with low vision and visual hallucinations who wish to focus on symptom-based care benefit greatly from their services.

## Conclusions

In this review, we present a case of Charles Bonnet syndrome in the setting of bilateral bacterial endophthalmitis and subretinal abscesses secondary to MRSA bacteremia. Throughout the hospital course, the patient developed both complex and simple hallucinations. Given the patient's poor visual acuity and normal neurologic and psychiatric work-up, the patient was diagnosed with Charles Bonnet syndrome. The combination of Charles Bonnet syndrome and subretinal abscesses is an atypical presentation for endogenous endophthalmitis. This case study not only provides a unique insight into a rare condition but also opens further insight into how we approach patients with low visual acuity, underscoring its potential implications for palliative patient care and the need for further research.
